# Strain localisation and failure at twin-boundary complexions in nickel-based superalloys

**DOI:** 10.1038/s41467-020-18641-z

**Published:** 2020-09-29

**Authors:** Zhenbo Zhang, Zhibiao Yang, Song Lu, Allan Harte, Roberto Morana, Michael Preuss

**Affiliations:** 1 School of Materials, University of Manchester, M13 9PL Manchester, UK; 2 Center for Adaptative System Engineering, School of Creativity and Arts, ShanghaiTech University, Shanghai, 201210 China; 3 Applied Materials Physics, Department of Materials Science and Engineering, Royal Institute of Technology, Stockholm, SE 100 44 Sweden; 4 Shanghai Key Laboratory of Advanced High-Temperature Materials and Precision Forming, School of Materials Science and Engineering, Shanghai Jiao Tong University, Shanghai, 200240 People’s Republic of China; 5 BP Exploration Operating Company Limited, Chertsey Road, Sunbury-on-Thames, TW16 7LN Sunbury, UK

**Keywords:** Mechanical engineering, Mechanical properties, Metals and alloys

## Abstract

Twin boundaries (TBs) in Ni-based superalloys are vulnerable sites for failure in demanding environments, and a current lack of mechanistic understanding hampers the reliable lifetime prediction and performance optimisation of these alloys. Here we report the discovery of an unexpected γ″ precipitation mechanism at TBs that takes the responsibility for alloy failure in demanding environments. Using multiscale microstructural and mechanical characterisations (from millimetre down to atomic level) and DFT calculations, we demonstrate that abnormal γ″ precipitation along TBs accounts for the premature dislocation activities and pronounced strain localisation associated with TBs during mechanical loading, which serves as a precursor for crack initiation. We clarify the physical origin of the TBs-related cracking at the atomic level of γ″-strengthened Ni-based superalloys in a hydrogen containing environment, and provide practical methods to mitigate the adverse effect of TBs on the performance of these alloys.

## Abstract (web summary)

Coherent twin boundaries in nickel-based superalloys are vulnerable sites for alloy failure in demanding environments. Here, the authors show that the abnormal γ″ precipitation mechanism at twin boundaries is responsible for pronounced strain localisation and subsequent failure.

## Introduction

Ni-based superalloys are widely used as structural components in a variety of highly demanding environments, such as in aerospace turbine engines, nuclear power plants and oil and gas deep wells^1–4^. For polycrystalline alloys serving in aggressive environments, intergranular degradation is known to be their Achilles’ heel for materials failure, which stimulates the development of grain boundary engineering (GBE)^5,6^. Accordingly, intergranular cracking can be alleviated in many materials by introducing a high proportion of coincidence-site-lattice (CSL) boundaries^7,8^. In contrast, as the typical CSL boundaries preferred under the framework of GBE, coherent twin boundaries (TBs) in some Ni-based superalloys are found to be vulnerable sites for materials failure^9–13^. Current lack of mechanistic understanding in the TBs-related failure makes it difficult to meet the failure-intolerant demands when Ni-based superalloys are used as critical components in various environments. The present study is therefore dedicated to achieve a bottom-up understanding in the origin of TBs-related failure in Ni-based superalloys.

Polycrystalline Ni-based superalloys inherently have a high proportion of coherent annealing TBs, due to their low stacking fault energy^14,15^. TBs in metals and alloys are known to be of merit for materials performance on various aspects^5,16,17^. Specifically, similar to general grain boundaries (GBs), TBs act as barriers to dislocation motion, which enhances the materials strength^16^; different from general GBs, they have a low excess free volume and a high surface separation energy, so they are supposed to be more resistant to intergranular cracking^17,18^. However, TBs in some Ni-based superalloys strengthened with γ′ phase are reported as promoters of crack initiation during fatigue loading^9,10^. Stinville et al. suggested that the elastic anisotropy across TBs, which triggers localised plasticity at TBs during cyclic straining, plays a significant role in fatigue crack initiation^10,11^.

In terms of environment-assisted failure, TBs are expected to be more resistant to hydrogen (H) assisted cracking due to their higher resistance to H segregation compared to general GBs^18,19^. Ingress of H can cause severe loss of ductility and sudden fracture of metals and alloys, which is known as H embrittlement (HE)^20^. It was indeed reported that the HE resistance of pure Ni benefits from introducing more TBs^21^. However, distinct roles of TBs in HE are found in Ni-based superalloys, and recent work by Seita et al indicated that H-induced cracks tend to initiate from TBs in a γ″-strengthened Ni-based superalloy^12^, which is provisionally suggested to be attributed to the H-enhanced dislocation plasticity and H-dislocation interplay along TBs.

Although the unexpected TBs-related failures have shown to be critical for the performance of Ni-based superalloys, the physical origin of its mechanism is still elusive. Heretofore, all studies rely on macroscale or microscale analysis^10,12,22^, without providing sufficiently detailed characterisations that can truly link the crack formation with TBs, let alone the corresponding atomic level origin. Moreover, as the main difference to pure Ni, the precipitates in superalloys (γʹ and γʺ), particularly at TBs, have not yet been considered in previous studies, which might well be the reason for the confusion around failure at TBs in Ni-based superalloys.

In this study, we conduct thorough multiscale microstructural and mechanical characterisations, coupled with density functional theory (DFT) calculations, on the TBs and TBs-related failure in γ″-strengthened Ni-based superalloys. We discover a TB complexion-mediated precipitation mechanism of γ″ phase, which leads to the formation of γ″ with distinctive morphology along TBs. Such special γ″ precipitate and the resulting precipitates denuded zone takes the responsibility for the premature dislocation activities and pronounced strain localisation during mechanical loading. With these findings, we uncover the physical origin of TBs-related failure on atomic level of Ni-based superalloys, in particular the H-induced crack initiation at TBs. We also demonstrate that H-induced cracks do not initiate exactly along TBs, but along the strain localised planes (a few tens of nanometers away from TBs), thereby clarifying the confusion surrounding the TBs-related failure. Based on these mechanistic understandings, we successfully mitigate the adverse effect of TBs in Ni-based superalloys by tailoring γ″ precipitates via a designated thermomechanical processing.

## Results

### Twin boundary and precipitate in superalloys

**Fig. 1:**
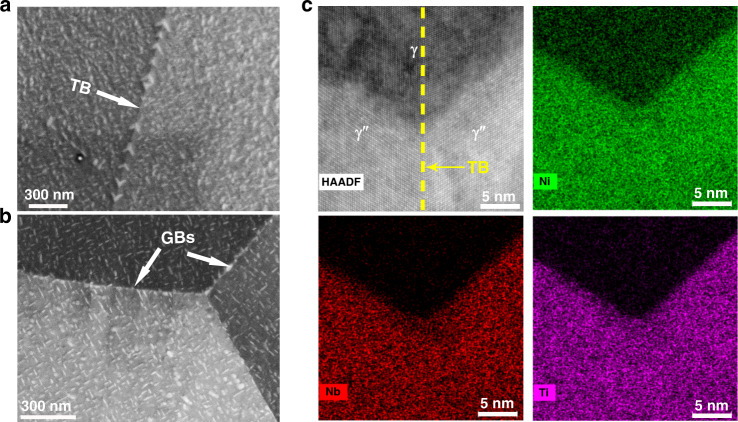
Morphology of γ″ precipitates at twin boundaries (TBs) and near general grain boundaries (GBs), and element distribution across TBs. BSE images showing (**a**) V-shaped γ″ along a twin boundary (TB) and (**b**) γ″ near GBs at a triple junction; **c** HAADF-STEM image containing γ and γ″ across a TB and the corresponding EDX elemental maps (Ni, Nb, Ti).

We conducted multiscale characterisation on the TBs in Alloy 945X (see methodology section for alloy composition), and 57.7% of GBs are coherent TBs (See Supplementary Fig. 1a,b). It is known that lens-shaped γ″ has a constant cuboid-on-cuboid orientation relationship (OR) with the γ matrix, i.e. (001)γ″ || (001)γ and [001]γ″ || [001]γ. γ″ precipitates across TBs inherit this OR, and they are therefore configured with a TB-related mirror symmetry when viewing along the [110]γ zone axis (See Supplementary Fig. 1c,d). Backscattered electron (BSE) image in Fig. 1a clearly shows symmetric V-shaped γ″ with one-to-one correspondence along the TB, while such morphology does not form at general GBs as seen in Fig. 1b. In addition, it is evident that γ″ precipitates along TBs are coarser than those in the grain interior. High resolution HAADF-STEM image with corresponding EDX elemental maps (Fig. 1c), demonstrate no detectable elemental depletion or enrichment along the TB of γ/γ and TB of γ″/γ″.

### Twin boundary complexion

To clarify the physical origin of the distinctive γ″ along TBs, namely coarser in size than the γ″ in the grain interior and symmetric one-to-one correspondence across TBs, atomic resolution Z-contrast imaging was performed using aberration-corrected scanning transmission electron microscopy (STEM).

**Fig. 2:**
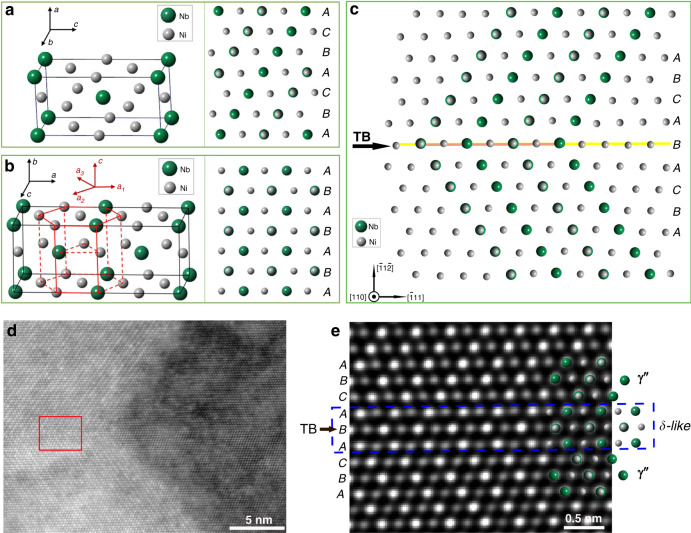
Atomic structure and crystal models of D0_22_-Ni_3_Nb γ″ and D0_a_-Ni_3_Nb δ precipitates, and the atomic structure of the precipitate at TBs. **a** Unit cell of D0_22_-Ni_3_Nb γ″ and atomic structure of γ″ viewing along [110] direction with *ABCABC* packing sequence; **b** two orthorhombic cells of D0_a_-Ni_3_Nb δ and its relationship with a hexagonal close-packed unit cell and atomic structure of δ viewing along [100] direction with *ABAB* packing sequence; **c** schematic crystal models of a TB with V-shaped γ″; **d** an HAADF STEM image showing the atomic structure of two γ″ across a TB with incident electron beam along [110] zone axis; **e** the corresponding IFFT image of the framed region in (**d**), and crystal models of γ″_[110]_ and δ_[100]_ are overlapped on part of the atomic image.

γ″ has a D0_22_ structure based upon the Ni_3_Nb^23^, as shown in Fig. 2a. The close-packed plane of γ″ is (112), which corresponds to the (111) plane of the face centred cubic (*fcc)* γ matrix. Similar to the *fcc* γ, the packing sequence of γ″ can be viewed along [1-10], and the schematic crystal structure of [1-10]_γ″_ is shown in Fig. 2a. HAADF-STEM image in Fig. 2d shows the atomic structure containing TBs both between γ″ and γ″ (γ″-TB-γ″) and between γ and γ (γ-TB-γ). The incident electron beam is along [110]_γ_, and the observed atomic structure of γ″ is consistent to the schematic atomic model in Fig. 2a, with *ABCABCABCA* packing order. With a TB embedded, the sequence of close-packed planes is *ABC****ABA****CBA*, and a …***ABA***…type sequence is encountered across the TB, as illustrated in Fig. 2c. This local packing sequence seems to be in accordance with the packing sequences of δ-Ni_3_Nb phase, which is the equilibrium phase of γ″-Ni_3_Nb. δ phase has an orthorhombic D0a structure^24^, and two unit cells of δ are presented in Fig. 2b. This D0a structure can be alternatively described as a hexagonal closed-pack structure, as illustrated in Fig. 2b. In this manner, the close-packed planes are (010), and the atomic arrangement of the (010)_δ_ plane is identical to the close-packed (112) plane of metastable γ″^24^. The basic difference between these two structures lies in the stacking sequence, and the close-packed plane of δ phase has the *ABABA* type stacking sequence, which can be visualised along the [100]_δ_ zone axis (Fig. 2b). In this context, a speculation naturally comes out: is the *ABA* type structure across a TB observed in the HAADF-STEM (Fig. 2c, d) identical to the structure of δ phase? Hence the atomic models of [100]_δ_ and [110]_γ″_ were compared with the atomic structure of the γ″-TB-γ″, as shown in Fig. 2d, e. Remarkably, the three *ABA* atomic layers can perfectly match the crystal structure of δ phase, while the adjacent regions match the γ″ phase (Fig. 2e). Since δ is a thermodynamically stable phase of γ″, formation of an *ABA* structure, i.e. precipitation of V-shaped γ″ along the TB, very likely is energetically favourable.

In order to further underpin this finding and uncover the related physical origin, DFT calculations were performed to assess the boundary energy of the TB between the V-shaped γ″ (details about DFT calculation are provided in the Method and Supplementary Note 1). We find that the TB boundary energy of the V-shaped γ″ is extremely low (0.6 mJ m^−2^), which represents a significant reduction from the coherent TB energy of the γ matrix (10.6 mJ m^−2^). Hence, precipitation of γ″ in V-shaped morphology across TBs reduces the boundary energy of TBs in the matrix, i.e. formation of V-shaped γ″ along TBs has a strong energetic preference compared to precipitating γ″ in the grain interiors (the average boundary energy between γ″ and γ is 76 mJ m^−2^). Consequently, γ″ is prone to nucleate at TBs at an earlier stage during heat treatment than those in the grain interior, which leads to coarser γ″ along TBs as the heat treatment proceeds. Our DFT calculations and experimental results also indicate that preferential γ″ nucleation is always in a one-to-one correspondence (γ″-TB-γ″), rather than in the morphology of γ″-TB-γ, because the latter results in a boundary with very high interfacial energy. Therefore, the atomic characterisation and DFT calculations demonstrate that the physical origin of preferential precipitation of V-shaped γ″ across TBs is the formation of δ-like tri-layer complexion across TBs with intrinsically low energy.

### Response of twin boundary during mechanical loading

**Fig. 3:**
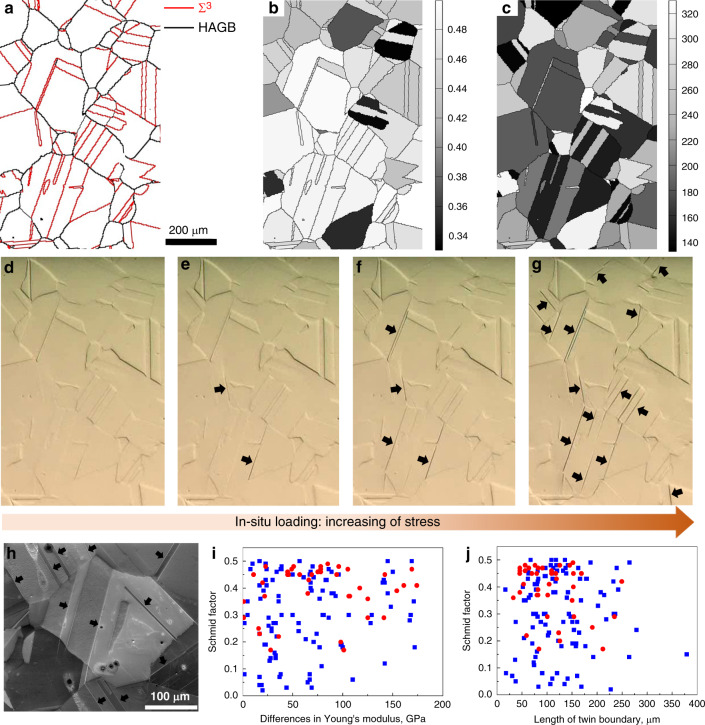
In-situ observation of the development of slip traces at TBs during tensile loading and statistical analysis of slip traces generated at TBs. **a**–**c** Microstructural information of a region of interest tracked during in situ loading, **a** grain boundary map obtained by EBSD; **b** Schmid factor map; **c** Young’s modulus map with unit in GPa; **d**–**g** snapshots taken during in situ tensile loading and visible slip traces are indicated by black arrows, and tensile axis is horizontal; **h** a SE image of a region from the sample after tensioning to a strain of 1%, and slip traces along TBs are indicated by black arrows; **i**, **j** Statistics of 150 TBs which have developed slip traces (red solid circles) and have not developed slip traces (blue solid squares) in a sample after tensioned to a strain of 1%, **i** diagram of Schmid factor versus the differences in Young’s modulus across TBs; **j** diagram of Schmid factor versus the length of TBs.

To investigate whether the distinctive V-shaped γ″ will affect the behaviour of TBs, in situ tensile test under an optical microscope was carried out to track the initiation and development of dislocation slip bands (DSBs) in Alloy 945X. Fig. 3a–c show the microstructural information of a region tracked during in situ test, including the grain boundary map (Fig. 3a), Schmid factor map (Fig. 3b) and Young’s modulus map (Fig. 3c). Fig. 3d–g show the snapshots taken during in situ loading (See Supplementary Movie1 for more details), where DSBs can be clearly identified. It is seen that DSBs initiate from the grains with high Schmid factors, and more DSBs appear with increasing of stress. Astonishingly, all the DSBs generated during this early stage are associated with TBs, as shown in Fig. 3g, whilst no DSBs were detected in the grain interior. This indicates that the critical stress for DSBs initiation at TBs is lower than that in the grain interior.

To reveal whether the premature dislocation activities at TBs have potential dependency on the characters of TBs, we have performed comprehensive statistical analysis (from a much larger area than Fig. 3a, shown in the Supplementary Fig. 2) on the characters of TBs, including the length and maximum Schmid factor of TBs, and elastic anisotropy across TBs. Fig. 3i and j show the plots of TBs characters separately for the TBs with and without DSBs developed. It demonstrates that formation of DSBs has no dependency on the elastic anisotropy across TBs and the length of TBs, and an example is presented in the Supplementary Fig. 3. The only factor that affects the tendency of DSBs formation along TBs is the maximum Schmid factor of the TBs. Accordingly, if a TB is oriented with a high Schmid factor with respect to the loading axis, DSBs will have high tendency to develop at the TB at relatively low stress, irrespective of the elastic anisotropy across the TB and the length of the TB.

**Fig. 4:**
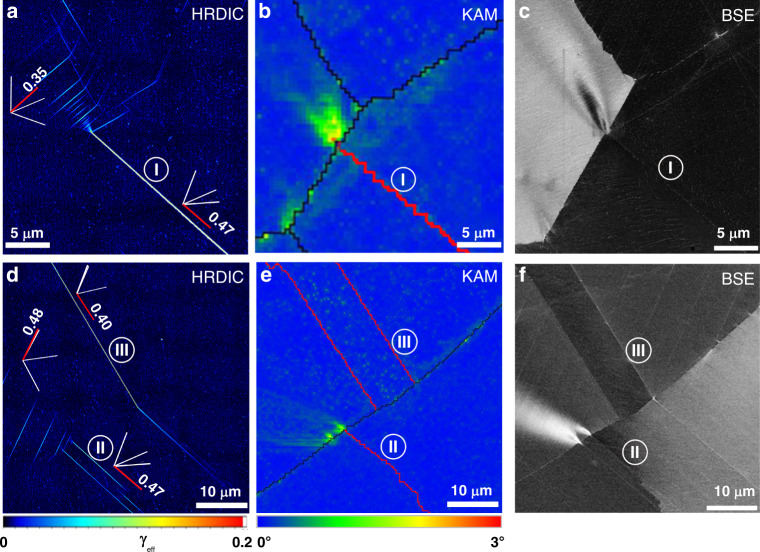
Strain localisation associated with TBs: Three TBs in two regions are presented to illustrate the strain localisation along TBs, misorientation gradient and volumes with high strain at grain boundaries. **a**, **d** Effective shear strain maps obtained by high resolution digital image correlation (HRDIC), where the four {111} plane traces are presented and the ones parallel to the TBs are coloured in red with their Schmid factors noted, and the colour scale of strain maps is presented below (**d**); **b**, **e** Kernel Average Misorientation (KAM) maps, and the colour scale of misorientations is presented below (**e**); **c**, **f** backscattered electron (BSE) images.

**Fig. 5:**
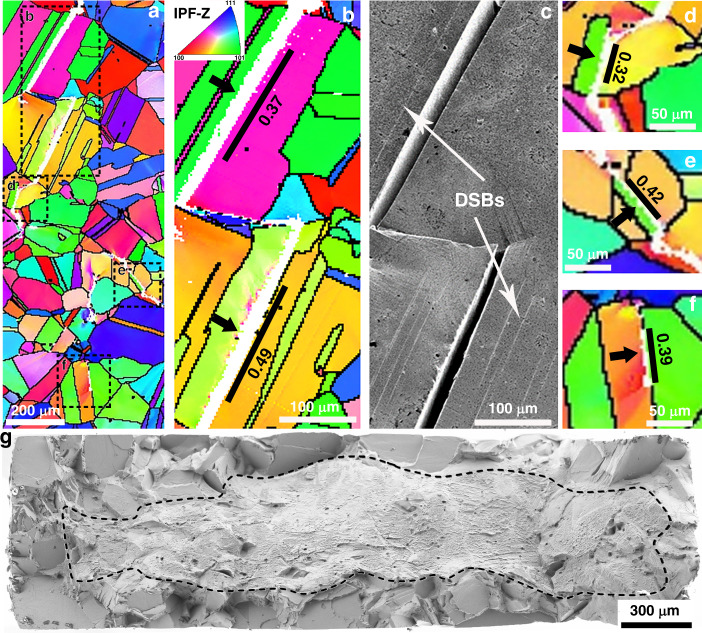
H induced cracking and failure associated with TBs in H-charged sample. **a** Orientation map obtained by EBSD on the sample surface after SSRT to failure; **b** a magnified image from (**a**) showing long cracks associated with TBs, which are indicated by black arrows, and Schmid factor of the TBs are noted; **c** the corresponding secondary electron image of (**b**) with dislocation slip bands (DSBs) indicated; **d**–**f** more magnified images from the framed regions in (**a**) to show cracks along relatively short TBs and extended to the neighbouring grains; The inset in (**b**) is the colour legend of all orientation maps. **g** A SEM image of the fracture surface showing very flat facets in the quasi-cleavage regions, where H was present during SSRT.

Premature dislocation-based plasticity at TBs during mechanical loading leads to very pronounced strain localisation. High-resolution digital image correlation (HRDIC) was employed to record the strain distribution in the sample tensioned to a total strain of 0.6%, where high strain localisation was only observed at TBs (See Supplementary Fig. 4). Fig. 4 shows the detailed microstructure and shear strain maps associated to three TBs with high Schmid factors. In addition to the strain localisation affiliated with the TBs, dislocation activities at TB I and TB II also lead to pronounced strain localisation at the intersection sites between the TBs and GBs, as shown in the Kernel Average Misorientation (KAM) maps (Fig. 5b and  e) and BSE images (Fig. 5c and f).

### Hydrogen assisted cracking at twin boundary

Slow strain rate tensile (SSRT) tests were conducted on the H-charged samples to evaluate the H effect on the materials performance. Compared to the non-charged sample, the elongation to failure of the H-charged sample was reduced from 18% to 2%. Fig. 5 shows EBSD orientation maps from a sample after SSRT to failure. Many H-induced cracks were detected at TBs, as shown in Fig. 5b–f. It should be noted that most of the cracks do not only initiate from TBs but also propagate to the adjacent grains. It is also evident that cracks form at TBs when they are oriented with high Schmid factors and DSBs generate on the planes parallel to TBs, irrespective of the lengths of TBs. Although micro volumes with high strain concentration can form due to the obstruction of slip transmission at GBs (Fig. 4), no crack initiation was observed from these sites, which is different to the crack initiation during fatigue in some superalloys^25,26^. The fracture surface of the region with H in presence is dominated by very flat facets, as seen in Fig. 5g. To determine whether these facets result from cracking along TBs, multiple methods were employed to characterise the facets. Confocal laser microscopy was used to determine the spatial orientation of the facets with respect to the sample geometry, and the inclined angles of the facets to the loading axis were then obtained. In addition, the orientations of the grains where the facets had formed were acquired by EBSD from the sample surface, and the four {111} plane traces and the inclined angles to the loading axis of each plane were calculated. Examples of four facets are shown in Supplementary Fig. 5. The data shows very good agreement in the inclined angles of the facets obtained by two methods, which proves that the facets are indeed along TBs.

### New mechanism for strain localisation and H-assisted cracking along twin boundary

**Fig. 6:**
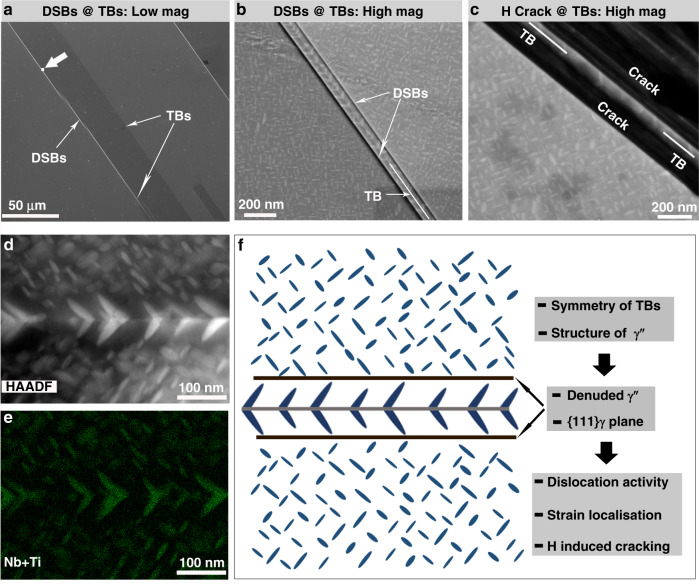
Multiscale analyses illustrate the mechanistic origin of strain localisation and H-induced cracks associated with TBs. **a** A secondary electron image showing a region with several TBs and dislocation slip bands (DSBs) at TBs from the sample after slow strain rate test (SSRT) to a strain of 1%; **b** a BSE image taken at very high magnification from the region masked by a white square in (**a**), where DSBs can be seen at the two sides of TB; **c** a BSE image taken at very high magnification from the H-charged sample after SSRT to failure showing two parallel cracks on both sides of the TB; **d**, **e** HAADF STEM image containing a TB and its corresponding EDX Ti+Nb elemental map; **f** a schematic diagram showing the configuration of γ″ around a TB, and coarser and V-shaped γ″ along a TB leads to formation of ‘weak’ planes with denuded γ″, which serve as easy channels for dislocation activities.

Current knowledge about TBs cannot explain the above abnormal behaviours related to TBs in Alloy 945X, namely very pronounced strain localisation, in the form of premature activation of dislocations during loading, and substantial H-induced cracks associated with TBs. To excavate the root cause, slip localisation and H-induced cracking were explored down to much finer scale. Fig. 6a shows a SEM image of a region with several TBs from a sample after tensioning to 1%, and DSBs along TBs are evident. Inspecting with a much higher resolution in BSE mode (Fig. 6b), it is surprisingly found that two DSBs form symmetrically along the planes about 40 nm away from both sides of the TB, instead of having a single DSB along the TB. These planes are parallel to the TB, and they are defined by the cusps of the V-shaped γ″ along the TB. Similarly, we re-examined the H-induced cracks at a much higher magnifications in BSE mode. Owing to the special configuration of γ″ along TBs, the exact location of TBs can be unambiguously identified. As shown in Fig. 6c, the cracks are not along the TB either, but they are along the planes about 40 nm away from the TB, which is identical to the location where DSBs developed (more direct evidences are present in Supplementary Fig. 6). Analogous to the DSBs, two cracks form parallelly along both sides of the TB.

These new findings suggest that strain localisation and H-induced cracking likely originate from the coarser V-shaped γ″-affected zones which form very adjacent to TBs. More detailed analysis about the γ″ at TBs was performed in order to fully address the issues related to TBs. Since TBs are the preferential sites for γ″ nucleation, the γ″ forming elements in the region near TBs tend to incorporate into γ″ precipitation along TBs, and such earlier nucleated γ″ has the advantage to grow at the expense of the surrounding γ″, which leads to the formation of regions on both sides of TBs with very few fine γ″. STEM-EDX analysis of a TB in Fig. 6d,e demonstrates that Nb and Ti are depleted in the region adjacent to the V-shaped γ″ and there is clearly a γ″-denuded zone. This leads to the formation of two special planes, which are defined by the cusps of V-shaped γ″ at TBs, as illustrated in Fig. 6f. Due to the highly symmetric configuration of γ″ along the TB, these two planes are parallel to the TB, i.e. these planes are parallel to {111} crystallographic planes. It has to be noted that in the present study we deliberately used a microstructural condition with slightly coarser γ″ to unambiguously elucidate the formation of V-shaped γ″ along TBs and its direct role on slip localisation and H-induced cracking. In fact, the V-shaped γ″ formed in all microstructural conditions (as illustrated in Supplementary Figs. 7, 8, and 11), because it originates from the intrinsic interplay between γ″ and coherent TBs. Once the highly ordered V-shaped γ″ precipitates, two parallel planes with destructed precipitation strengthening from randomly-distributed γ″ will form, premature dislocation activities and strain localisation are inevitably incurred (evidences from the samples with different microstructural conditions are shown in Supplementary Figs. 8–10). Since the distance between the ‘weak’ plane and the TBs is determined by the size of the γʺ, one of the main differences between the sample in an under-aged condition (or faster cooled) with very fine precipitates and the sample in an over-aged condition with coarsened precipitates is that the spacing between the dislocation slip bands (DSBs) and the TBs is very small in the former case, and it is difficult to separate the DSBs from the TBs. An example from the under-aged sample is shown in Supplementary Fig. 14a, where DSBs are too close to the TBs, even though occasionally we can distinguish between them (see Supplementary Fig. 14b). The other difference is the stress difference in activation of DSBs near TBs and away from TBs. Since the ‘weak’ plane near TBs is almost free of precipitation strengthening, the difference in dislocation activation stress is solely dependent on the contribution from γʺ strengthening. Therefore, in the under-aged condition with smaller precipitates, precipitation hardening is not maximised in the matrix, so the stress difference in the activation of DSBs near TBs and away from TBs is smaller. In this manner, premature dislocation activity and strain localisation near TBs is supposed to be less pronounced, which will be explored later in tailoring the microstructure. In summary, these γ″-denuded zones are special in two aspects: (1) they are parallel to dislocation slip planes; and (2) they are weakened due to the lack of precipitation strengthening. Therefore, these planes can serve as easy channels for dislocation activation and slip, which leads to premature dislocation-induced plasticity and strain localisation associated with TBs during mechanical loading.

## Discussion

It is now clear that strain localisation and H-induced cracking at TBs originate from the distinctive γ″ along TBs. As the main strengthening factor, γ″ nucleates and grows during cooling after solution treatment and subsequent aging^27^. To clarify when the distinctive γ″ starts forming at TBs, a solution-treated sample was further characterised (see Supplementary Fig. 11). The V-shaped γ″ is evidently seen along TBs in this sample without aging, which indicates that such distinctive γ″ attributes to the nucleation process when the alloy was cooled after solution annealing. Due to the fact that γ″ is enriched in Nb and Ti, γ″ would have the preference in nucleation and growth along TBs if Nb and/or Ti segregate along TBs during solution treatment. Based on DFT calculations, Zhou et al.^28^ reported that TBs in alloys Inconel 718 and Inconel 725 have a much higher solubility in alloying elements (Nb, Ti, Al, and Cr) than the grain interiors. In addition, inspired by the concept of grain boundary complexion^29^, we suspected that TBs may have formed similar complexion with element segregation. To verify the calculation result and check whether or not there is any element segregation along TBs to assist the precipitate nucleation, we carried out high resolution EDS analysis at TBs in the alloy 945X after solution annealing. However, we found no detectable difference in alloying elements between the TBs and grain interiors (See Supplementary Fig. 12). This demonstrates that there is in fact no alloying element segregation along TBs. Therefore, we believe that it is the inherent crystal symmetry across a TB that provides a unique condition to locally form a more stable δ-like structure, which establishes the energetics preference for γ″ nucleation at TBs in a one-to-one correspondence manner. In analogous to grain boundary complexion, where elemental segregation usually results in a bilayer complexion at grain boundaries^30,31^, TBs in γ″-strengthened superalloy provides a trilayer structural complexion to promote the γ″ nucleation. Accordingly, the formation mechanism of V-shaped γ″ is of little dependence on the alloying elements, i.e. the V-shaped γ″ should universally exist in all γ″-strengthened superalloys, such as for example Inconel 718, Inconel 625, Inconel 725 or Incoloy 909. Indeed, we have also observed such special γ″ decorated TBs in another γ″-strengthened Ni-based superalloys, Inconel 718 alloy, as shown in Supplementary Fig. 13. Moreover, since the physical origin of the V-shaped γʺ is the inherent structure of D0_22_ precipitates and Σ3 coherent TB, the formation of V-shaped precipitates may exist in many other *fcc* alloys with D0_22_ type precipitates with much broader impacts, for instances, Al_3_Ti and Al_3_Nb in some Al alloys^32,33^, Cu_3_Sn precipitate in a Cu alloy^34^, and Ni_3_Mo in Ni-Mo alloys^35^.

Strain localisation associated with TBs was also reported to generate from the region adjacent to TBs instead of exactly along TBs in γʹ-strengthened Ni-based superalloys, and a strong dependence on the elastic anisotropy across the TBs was proposed to explain such strain localisation^10,11^. However, we find that the only factor determining the premature onset of localised slip at a TB in alloy 945X during mechanical loading is the Schmid factor of the TB. This phenomenon can be completely understood after uncovering the mechanistic origin of the strain localisation at TBs, as proposed above. Dislocation activities along the weak planes determined by the V-shaped γ″ is not supposed to be of any dependence on the TBs length and elastic anisotropy across the TBs. Whenever TBs are oriented for high Schmid factors with respect to the loading axis, premature dislocation plasticity will preferably operate along the weak planes in the fine γ″-denuded zones near the TBs.

Strain localisation is known to be detrimental for material properties in many aspects, such as uniform elongation^36^, fatigue life^37,38^, stress corrosion cracking resistance^39^, and H embrittlement resistance^40,41^. Our current work undoubtedly manifests that the strain localisation at TBs is the root cause for H-induced cracking from TBs. More importantly, we clarify that H-induced cracks associated with TBs are not exactly along TBs, but along the weak planes closely affiliated to TBs, where premature dislocation activities along these planes serve as the precursor for crack initiation. With this finding, we resolve the long-standing contradiction between the theoretical and experimental results on the role of TBs in H embrittlement: in theory, TBs have low H solubility and high surface separation energy compared to general GBs, so they should be more resistant to crack initiation^18,19^; in practice, TBs are found to be more vulnerable for crack initiation with H in presence^12^. This can be fully understood now because H-assisted cracking at TBs is essentially cracking along the very adjacent DSBs. H-induced cracking along DSBs has been well recognized in Ni-based superalloys^40–43^. DSBs are the locations with high strain field and high defect density, and H tends to gather at these sites^44–46^. H is known to be able to interplay with dislocations and other defects, which promotes voids formation and subsequent crack initiation^47–49^. This phenomenon becomes far more pronounced when dislocation activities are only along the weak planes associated with TBs at a stress level well below the expected yield stress. Consequently, H-induced cracks can initiate below a yield point is detected during mechanical loading, which was indeed observed in our previous research^50^. It should be pointed out that this phenomenon and underlying mechanism seems to be different to the one reported by Seita et al.^51^, where they proposed H-induced crack initiation along TBs in a Ni-based superalloy. As evidently shown in Figs. 6a–c, it is impossible to discriminate between cracking along TBs and cracking along the adjacent DSBs unless inspecting at sufficiently high magnifications, in particular in the sample with very fine γ″. We think that this is most likely the reason for the discrepancy between Seita’s work and our present work in identifying the exact cracking sites. In addition, Seita et al.^51^ reported that H-induced cracks are more reluctant to propagate along TBs, whereas we found that crack initiation along TBs also facilitates crack propagation. We indeed observed some short cracks that initiate along TBs and they are confined along those TBs, as shown in Fig. 5f, which seems to be similar to Seita’s claim. However, the majority of cracks not only initiate along TBs but also extend across several grain boundaries, as seen in Fig. 5b, d, e. This seems to be more rational, because crack initiation along TBs will in turn weaken the planes and facilitate crack growth during further loading. These cracks can propagate into neighbouring grains when they are long enough, or coalescence with other cracks along GBs/TBs, as seen in Fig. 5. Moreover, it is recognized that the degree of hydrogen embrittlement (HE) is often dependent on the H-charging conditions and the amount of hydrogen adsorbed by the alloy. Nevertheless, both Seita et al.’s work and our work are in fully agreement with the preferred H-induced cracking at TBs, which is such central aspect in order to understand HE in this class of alloys.

With such mechanistic understanding, we further explore possible strategies to alleviate the detrimental effect of TBs in Ni-based superalloys. We have found out that the pronounced strain localisation at TBs can be alleviated by minimising the size difference in γ″ between that along TBs and in grain interiors. This was achieved in Alloy 945X by applying a faster cooling rate after solution annealing and slightly lower aging temperatures. Relevant results are described in Supplementary Fig. 15. It shows that the difference in stress for dislocation activation at TBs and grain interiors becomes much smaller in the tailored microstructure, even though the critical stress for dislocation activation is still lower at TBs. Under such circumstances, TBs are much less vulnerable in terms of premature dislocation-induced plasticity and strain localisation, and strain is more evenly distributed across grains when subjected to mechanical loading. As a result, H-induced cracking along TBs is considerably mitigated and the susceptibility to hydrogen embrittlement is reduced, as shown in Supplementary Fig. 16.

In conclusion, the role of TBs in γ″-strengthened Ni-based superalloys is mediated by the distinctive γ″ along TBs. The inherent characters of γ″ and crystal-symmetry of TBs lead to the formation of a trilayer complexion at TBs facilitating γ″ nucleation and growth. Considering this phenomenon has little dependence on the alloy chemistry, V-shaped precipitates with D0_22_ crystal structure is likely to be a common feature in all γ′′ strengthened alloys. Our findings are significant in understanding and predicting the performances of γ″-strengthened Ni-based superalloys not only in hydrogenating environments, but also in more general applications where strain localisation plays an important role, such as stress corrosion cracking and fatigue. Moreover, our work provides a pathway to mitigate the adverse effect of TBs on the alloys performance in demanding environments by tuning the γ″ at TBs, which can be further explored to optimise the properties of Ni-based superalloys for specific applications.

## Methods

### Materials and microstructural characterisation

The majority of the present work was conducted on Incoloy Alloy 945X (UNS N09945). The alloy was supplied by Special Metals Wiggin Ltd as forged bars in solution-annealed and aged condition. The chemical compositions determined by X-ray fluorescence (XRF) are given in Table 1. Blanks machined from the ingot to a dimension of 12 × 12 × 60 mm were further heat-treated to boost the strength of the alloy. The blanks were solution-annealed at 1040 °C for 1 h and then cooled in the furnace with a cooling rate of 5 °C per minute. Subsequently, a two-stage aging treatment was carried out, via aging at 735 °C for 6 h and followed by aging at 635 °C for 18 h. To minimise the surface oxidation, all heat treatments were carried out in an argon atmosphere. After this heat treatment, the yield strength and total elongation of the alloys are 1178 ± 11 MPa and 23.4 ± 1.5%, respectively. This is the alloy condition used in most part of the current study unless other treatments are noted in the manuscript for specific purposes.

**Table 1:** Chemical composition of Alloy 945X determined by XRF.

Alloy	Ni	Cr	Mo	Nb	Ti	Al	Fe
945X	53.5	20.6	3.2	4.1	1.5	0.1	Bal.

Electron backscattered diffraction (EBSD) analysis was performed using a FEI Quanta650 field emission gun (FEG) SEM equipped with a HKL system operating at 20 kV. To image the very fine γ″ at TBs in bulk samples, high resolution backscattered electron (BSE) imaging was conducted on a FEI Magellan 400 FEG SEM equipped with a concentric backscatter electron detector. The microscope was operated at 1 kV with 0.1 nA in the immersion mode to achieve the required resolution and contrast. Samples for EBSD and BSE imaging were finished by polishing with 40 nm colloidal silica suspension (OPS, Struers).

Transmission electron microscopy (TEM) and scanning transmission electron microscopy (STEM) analysis on the twin boundaries and precipitates were conducted on probe-corrected FEI Titan G2 80–200 kV equipped with Super-X EDX detectors and Bruker Esprit software. High-resolution high angle annular dark field (HAADF) STEM images were acquired with a probe convergence angle of 21 mrad, an inner angle of 48 mrad and an outer angle of 220 mrad. A probe current of 90 pA was set for STEM-EDX spectral imaging. TEM foils were prepared by twin-jet electropolishing (TENUPOL5, Struers), in a solution of 10% perchloric acid and 90% methanol at a temperature of −35 °C.

### DFT calculation

The total energies for all the structures were performed by using the exact muffin-tin orbitals (EMTO) method based on the DFT^52–54^. The EMTO method is an improved screened Korringa-Kohn-Rostoker (KKR) method^55^, where the one-electron potentials are represented by large overlapping muffin-tin potential spheres. Generalized gradient approximation (GGA) parameterized by Perdew, Burke, and Ernzerhof (PBE) was used to treat the exchange-correlation functional^56^. In the self-consistent calculations, the one-electron equations were solved within the soft-core scheme and scalar-relativistic approximation. The Green’s function was calculated for 16 complex energy points distributed exponentially on a semi-circular contour, including the states within 1 Ry below the Fermi level. The irreducible parts of the Brillouin zones were sampled by uniformly distributed *k*-points. The *k*-mesh was carefully tested and the 12×24×3 mesh was adopted for the supercell calculations.

The chemical disorder was described with the coherent potential approximation (CPA) as implemented in the EMTO method^57^. CPA is an efficient and useful approach to treat the compositional and magnetic disorders. Spin-polarized calculations for Ni_3_Nb show very weak magnetic moments^58,59^, and the Curie temperature estimated by using a mean-field approach^60^ is ~23.5 K, indicating that it is paramagnetic (PM) for all temperatures of interest. The Ni-Fe-Cr matrix is also normally PM at temperatures higher than room temperature. Thus in the present study, all calculations were performed at the PM state, which was modelled by the disordered local moments (DLMs) method^61^.

The intrinsic stacking fault (ISF) in fcc structure was created by a rigid shift of one part of the fcc supercell along the 1/6[11-2] direction on the (111) slip plane. The stacking fault energy (SFE, *γ*_isf_) calculations were performed for a supercell containing 12 fcc (111) layers. The *γ*_isf_ of the D0_22_ structure was calculated in a similar way. Here, the Miller indices for both crystallographic directions and planes were represented in the basis of the fcc for convenience. Notice that in order to create the true ISF in the ordered structure, the shift vector is the 1/6[11-2] on the (111) close-packed plane^62^. Shift by 1/6[−211] would create the complex stacking fault. Twin is formed by having ISFs on consecutive close-packed planes. From the structural point of view, the twin boundary contains one hcp-like layer, while the ISF contains two, which forms the geometrical reason for the universal $\gamma _{tw} \approx 1/2\gamma _{isf}$ relationship.

### In situ tensile test

In situ tensile tests were conducted on a 5 kN Deben microtester with an initial strain rate of 1 × 10^−5^ s^−1^. Flat tensile specimens with a gauge length of 25 mm, a width of 3 mm, and a thickness of 1 mm were machined by means of electro-discharge machining. The machined samples were ground and polished with 40 nm colloidal silica suspension (OPS, Struers) to achieve mirror quality surface finish. Videos with a frame size of 1600 × 1200 pixels were recorded at 15 frames per second during loading, using a Keyence VHX-5000 optical microscope.

### H charging and slow strain rate test

H was introduced into the flat dog-bone samples by cathodic charging. Before charging, samples were ground and then polished in diamond suspension and colloidal silica suspension finishing with a quality for EBSD. It is known that H diffusion coefficient is very low in Ni-based superalloys^63^, and in order to introduce sufficient amount of H into the samples, the following charging protocol was used^40,43^. Cathodic charging was conducted at 80 °C in a solution with NaCl (1 mol L^−1^) and distilled water. The samples were charged with constant electric current density of 7.7 mA cm^−2^ for 168 h, using platinum foil as a counter electrode. The H penetration depth is estimated to be 150–200 μm on the sample surface, and the H concentration is around 70 ppm which is measured using the Eltra 900OH hydrogen analyser. After charging, the sample surface was polished with colloidal silica suspension for about 5 minutes to remove any contamination caused by charging.

Slow strain rate tensile (SSRT) tests were performed on the H-charged samples to evaluate the effect of H on the property degradation. SSRT was carried out in the air at the room temperature using an Instron machine with a 50 kN load cell with initial strain rate of 10^−6^ s^−1^. An extensometer was employed to measure the instant strain of the sample during tensioning. To minimise the H degassing, SSRT was done within 1 h after H charging. For comparision, a sample without H charging was tested in the same conditions. The fracture surface of samples after failure was characterised using a FEI Quanta 650 SEM. Topographical information of the fracture surface was obtained using a Keyence X200K 3D Laser Microscope.

### High-resolution digital image correlation (HRDIC)

HRDIC was used to record the strain developed in the sample during tensioning. The details about HRDIC experiment and analysis can be found in previously reports^64^. Generally, HRDIC was done via three steps: (1) sample preparation; (2) imaging before and after deformation; (3) data analysis. The flat dog bone sample (gauge geometry of 25 × 3 × 1 mm) for HRDIC was ground and polished with a quality for EBSD. Nanoscale gold particle patterns were put on the sample via gold sputtering and gold remodelling in water vapour at 315 °C. 25 × 25 BSE images with a single tile with of 25 µm were collected before and after tensile deformation from the same region using the FEI Magellan 400 FEG SEM. Tensile deformation was performed on a Instron machine with an initial strain rate of 1 × 10^−5^ s^−1^. An extensometer was used to measure the instant strain, and the sample was tensiled to a total strain of 0.6%. The BSE images were processed by La Vision DaVis software and a strain field map was then generated. Cross correlation of the images prior to and after deformation was done with a final interrogation window size of 8 × 8 pixels, which equates to a spatial resolution of about 97 × 97 nm.

## Supplementary information

Supplementary Information

Description of Additional Supplementary Files

Supplementary Movie 1

## Data Availability

The data that support the findings of this study are available in the 10.5281/zenodo.3964486 repository.
