# Supplementary material for: Strain localisation and failure at twin-boundary complexions in nickel-based superalloys
Source: Nat Commun. 2020 Sep 29;11:4890. doi: 10.1038/s41467-020-18641-z (PMC7524752; doi:10.1038/s41467-020-18641-z)
Supplement: Supplementary file 1 — Supplementary Information [file 41467_2020_18641_MOESM1_ESM.pdf]

# Supplementary Information

Strain localisation and failure at twin-boundary complexions in nickel-based superalloys

Zhang *et al.*

## Supplementary Figures

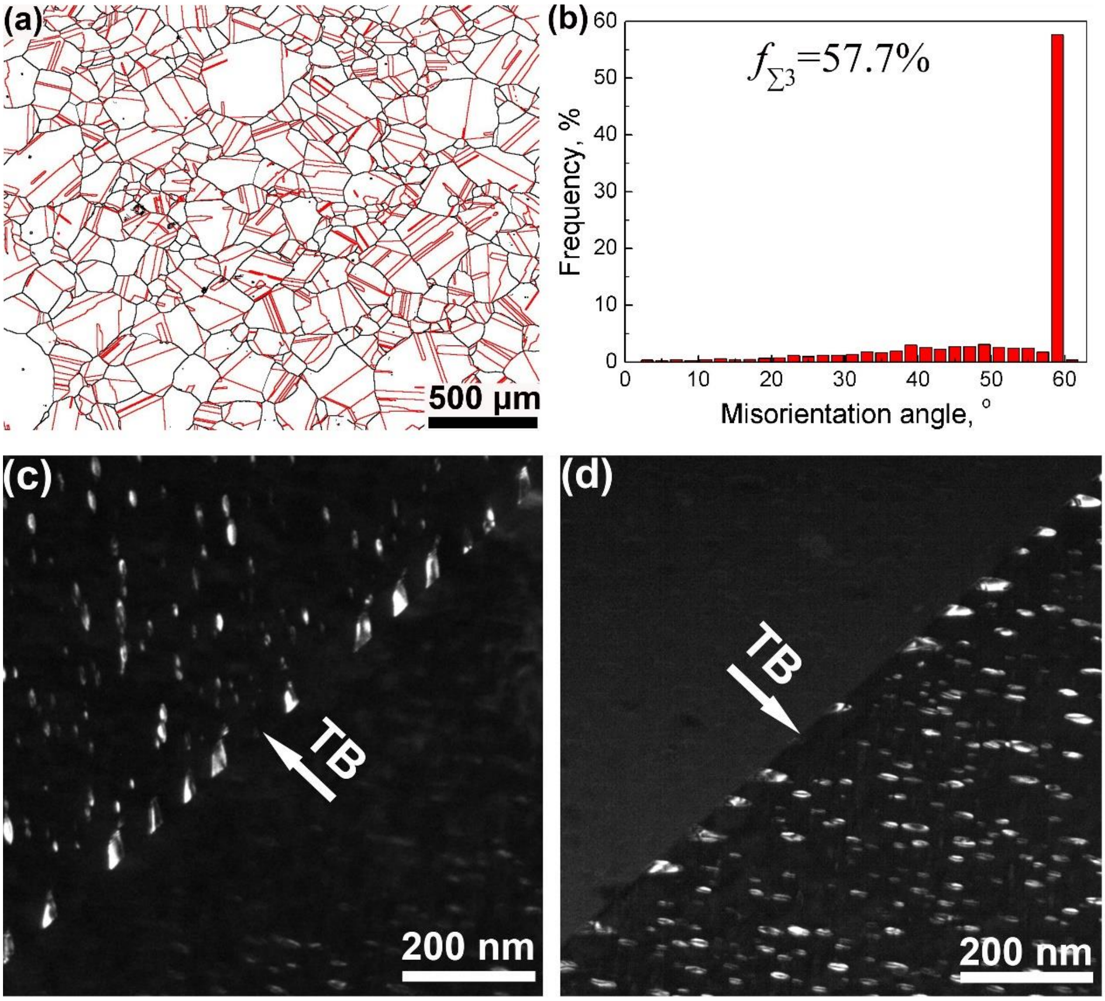


**Supplementary Figure 1.** Twin boundaries in Alloy 945X: (a) boundary map obtained by EBSD, where red lines and black lines indicate the Σ3 twin boundaries and general high angle boundaries, respectively; (b) misorientation angle distribution of all grain boundaries; (c) and (d) dark field TEM images of γ″ from the grains at both sides of the TB, incident electron beam is along <110>$_\gamma$

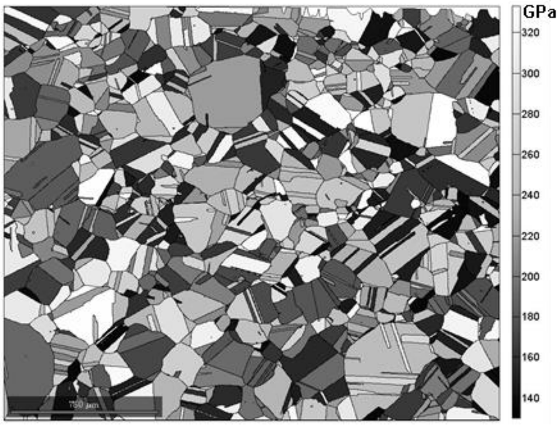


**Supplementary Figure 2.** Young's modulus map of alloy 945X that is used for statistic analysis in Fig.3i and 3j obtained from EBSD data.

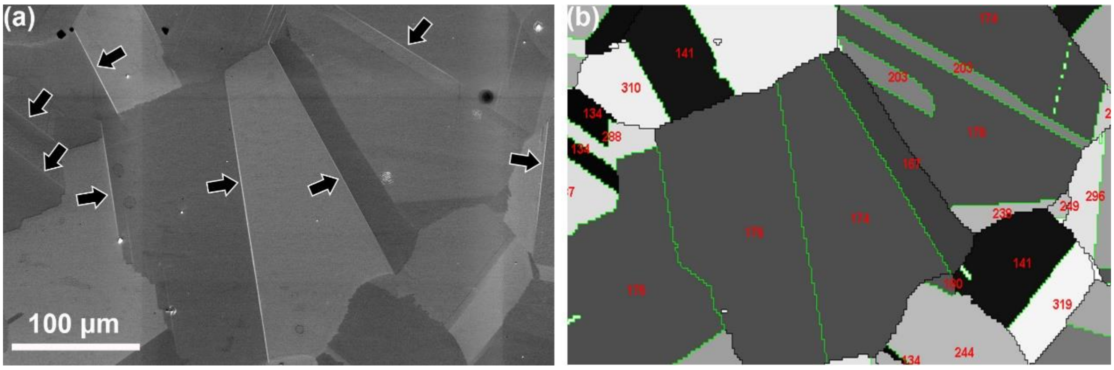


**Supplementary Figure 3.** (a) SEM image from a region in Figure R9 with slip traces along TBs indicated by arrows; (b) the corresponding elastic modulus map with the elastic modulus notated in each grains, and twin boundaries are highlight in green color

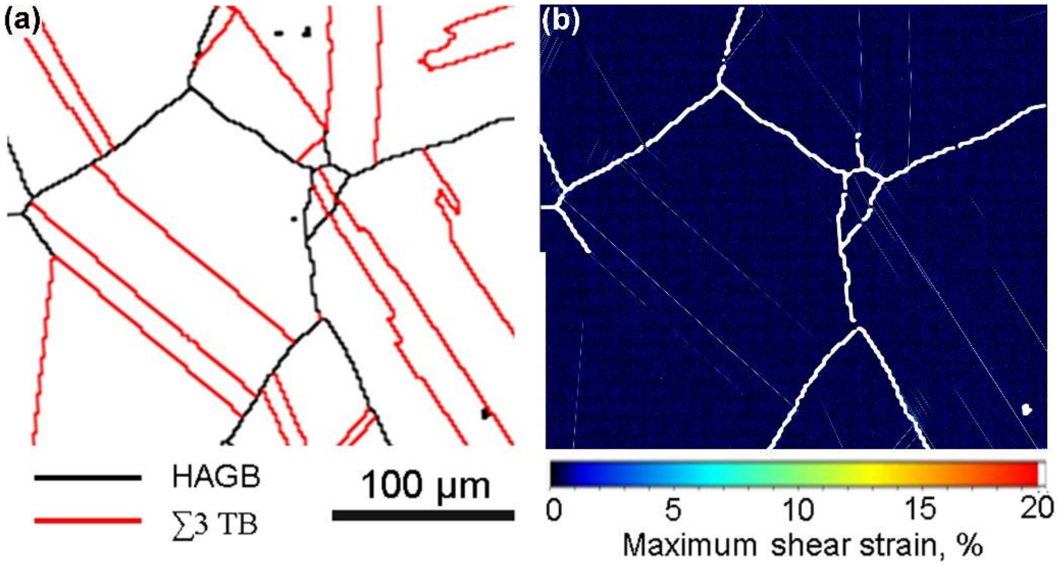


**Supplementary Figure 4.** HRDIC analysis of shear strain distribution in the sample after tensioning to a strain of 0.6%: (a) EBSD grain boundary map and (b) the maximum shear strain map obtained by HRDIC from the same region in (a) with the colour legend below (b)

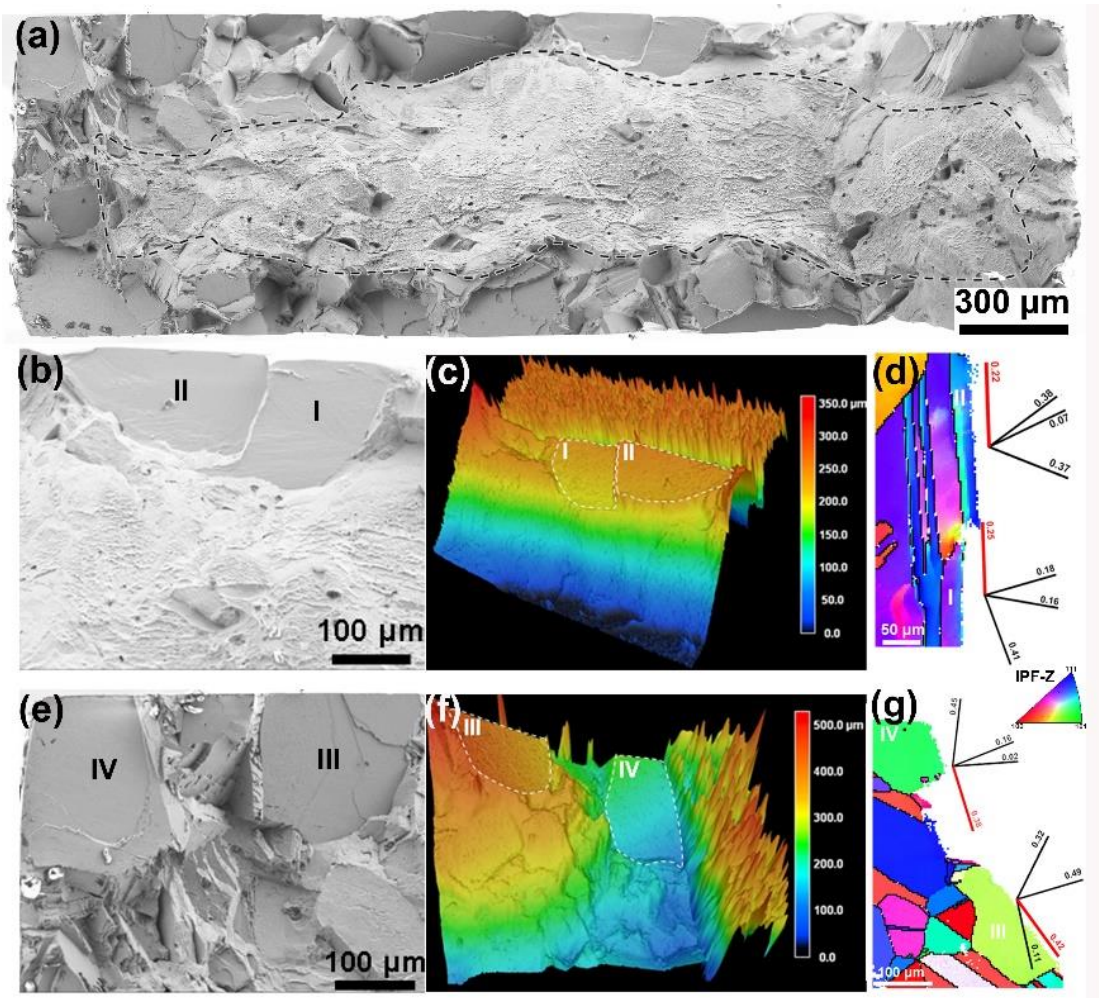


| Facet code | Plane trace | Inclined angle of (111) plane (EBSD) | Inclined angle of facet (Confocal Laser Microscope) |
|---|---|---|---|
| I | {111} | 73.4° | 71.7°±1.8° |
| II | {111} | 71.7° | 70.4°±1.5° |
| III | {111} | 90.5° | 92.4°±3.5° |
| IV | {111} | 67.1° | 68.2°±2.7° |

**Supplementary Figure 5.** Fractographic analysis of the H-charged Alloy 945X after SSRT to failure: (a) an overview SEM images of the whole cross section showing the quasi-cleavage fracture in the outer ring; (b) and (e) showing four flat facets selected from the quasi-cleavage region in (a); (c) and (f) the corresponding images obtained by confocal laser microscope; (d) and (g) EBSD maps from the sample surface containing the facets I, II, III and IV, the {111} plane traces and their maximum Schmid factors are noted. A table below the images summarises the data obtained from EBSD and confocal laser microscope.

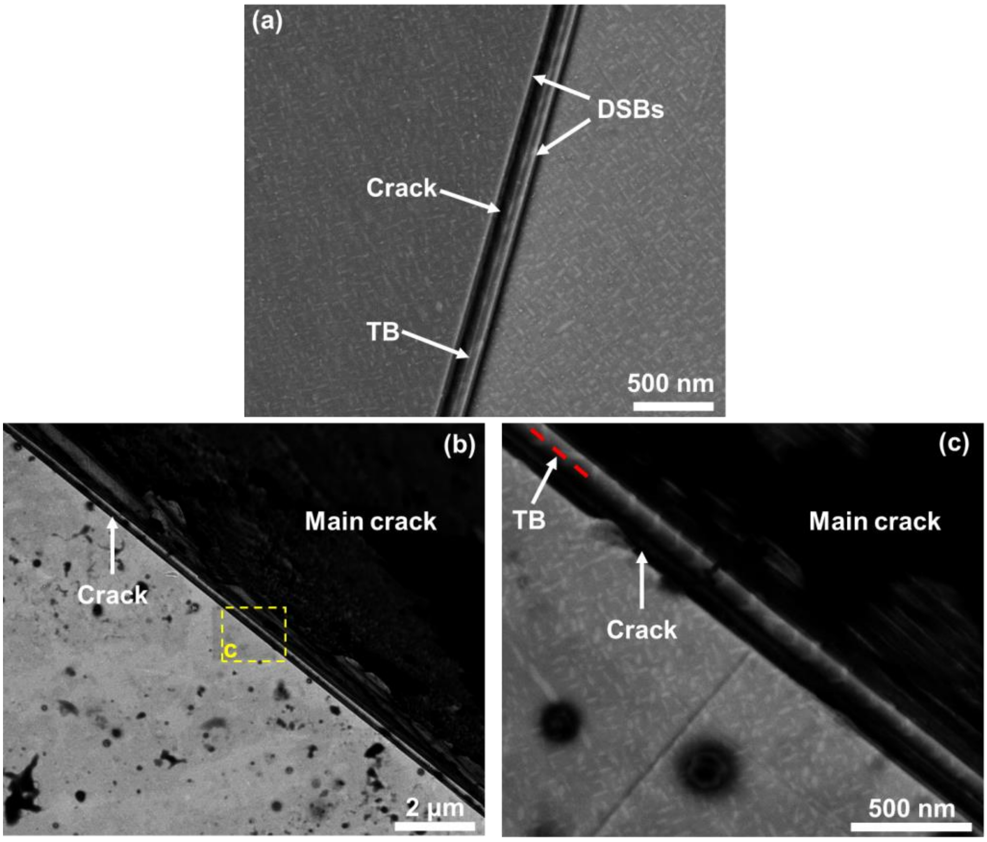


**Supplementary Figure 6.** BSE images showing H-induced cracking along the DSBs at the vicinity of TBs in Alloy 945X: (a) a crack along a DSB at a TB, which is in a region far from the fracture surface; (b) cracks near a TB close to the fracture surface; (c) an enlarged BSE image from (c) showing both the main cracks and a secondary crack exactly follow the DSBs developed due to the V-shaped γʺ.

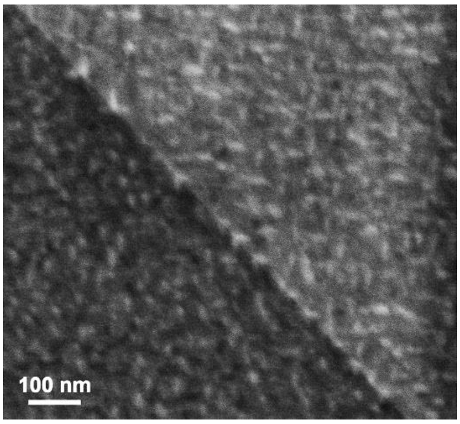


**Supplementary Figure 7.** BSE image showing γʺ along TBs and in the grain interior in an under-aged 945X sample (annealed at1040 °C/1h followed by cooling to ambient temperature with a rate of ~900 °C/h, and then aged at 735 °C/6 hrs + 635 °C/18 hrs)

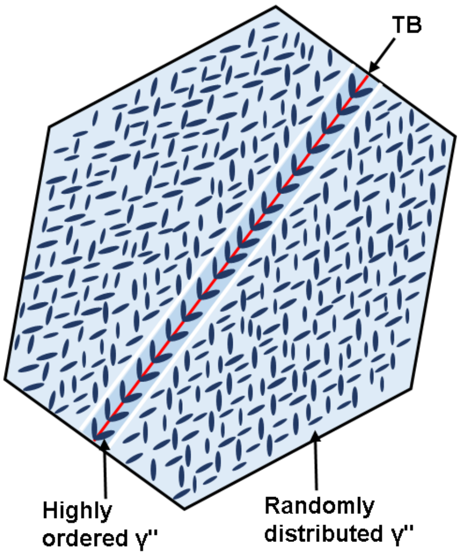


**Supplementary Figure 8.** Schematic diagram illustrates how the highly ordered γ″ along TBs incurs the formation of weak planes without precipitation strengthening.

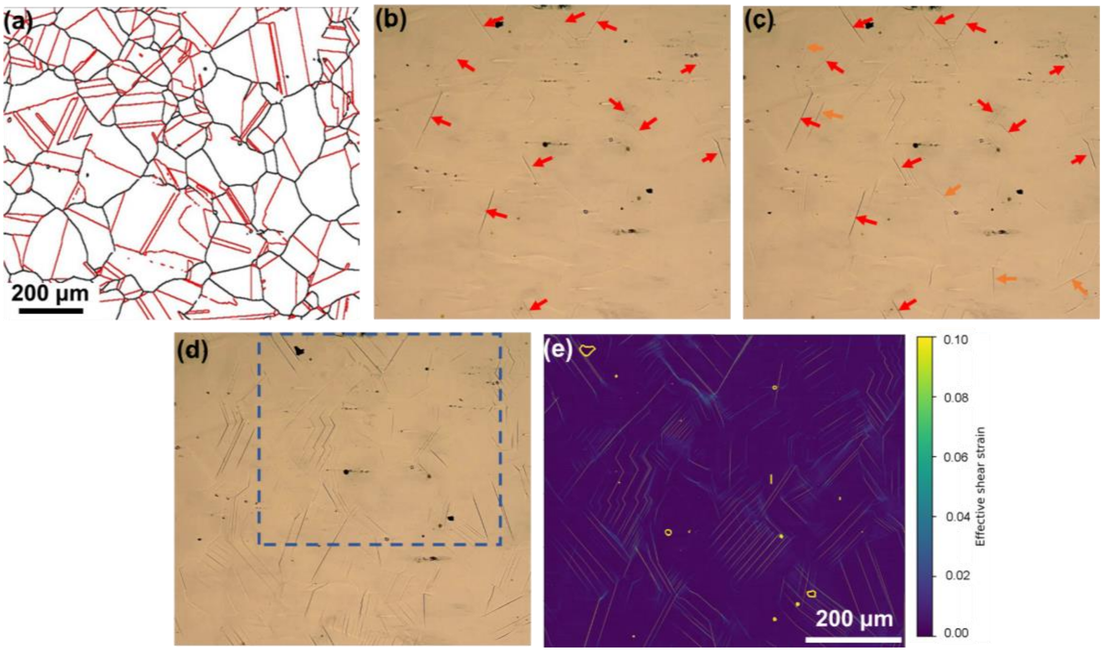


**Supplementary Figure 9.** Slip trace development captured during in-situ loading and strain map obtained by HRDIC: (a) grain boundary map obtained by EBSD with twin boundaries in red lines; (b)-(d) images from the identical region as (a) recorded during loading; (e) shear strain map obtained by HRDIC from the framed region in (d). The sample has the same condition as that in Figure R3 (annealed 1040 °C /1h, cooling in air; aged 706 °C/6 hrs + 607 °C/18 hrs).

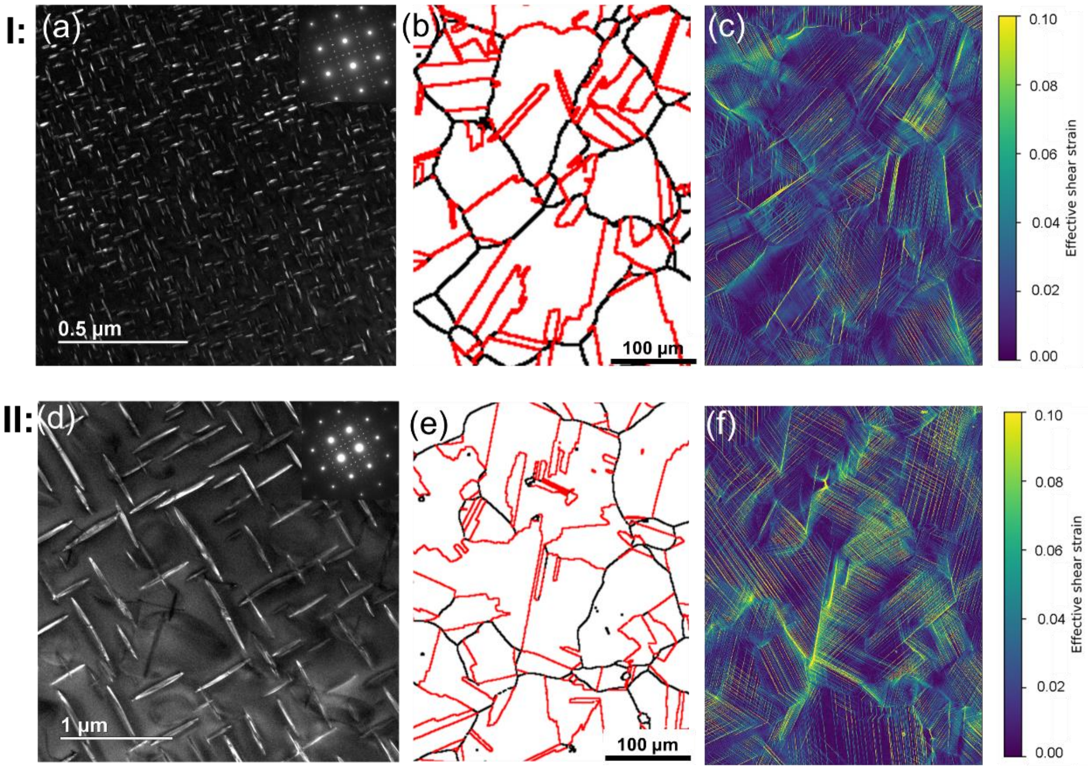


**Supplementary Figure 10.** Microstructure and shear strain maps of Inconel 718 alloy after different heat treatments (I) annealed 1040 °C /1h, air cooling and aged at 774 °C /6hrs and (II) annealed 1040 °C /1h, air cooling and aged at 800 °C /48hrs with different microstructures: (a),(d) dark field TEM image showing γ″ precipitates; (b), (e) grain boundary maps with TBs in red lines; (c), (f) shear strain maps from the same region shown in (b) and (e) respectively, of the two samples after tensioning to a total strain of about 2%.

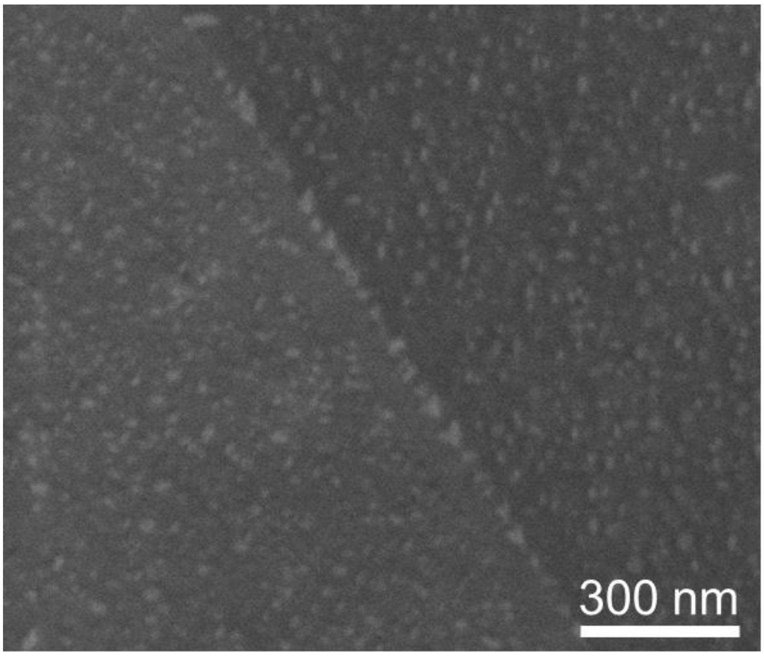


**Supplementary Figure 11.** BSE image of Alloy 945X after solution annealing at 1040 °C for 1 h followed by cooling to room temperature with a cooling rate of 5 °C/min

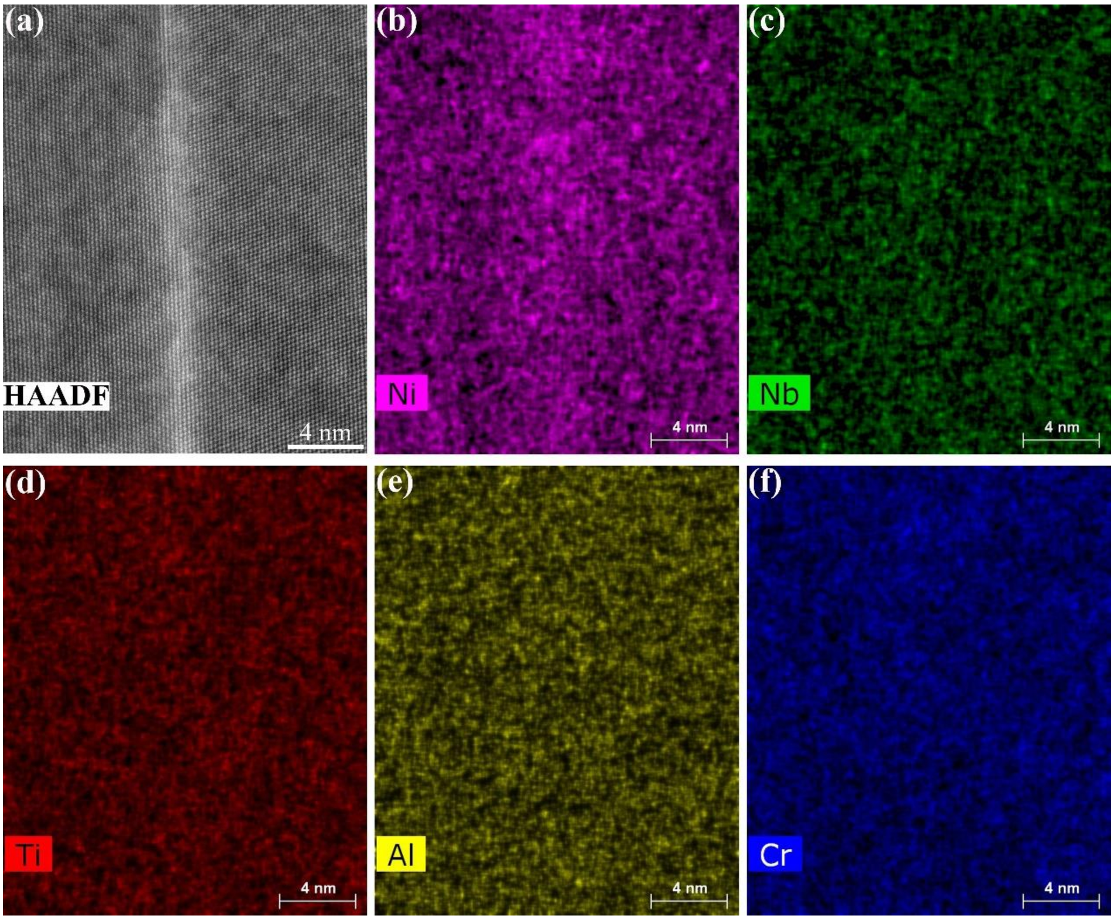


**Supplementary Figure 12.** STEM-EDX analysis of a TB in the Alloy 945X after solution annealing and water quenched: (a) HAADF STEM image containing a TB; and its corresponding EDX elemental maps (b) Ni; (c) Nb; (d) Ti; (e) Al; (f) Cr

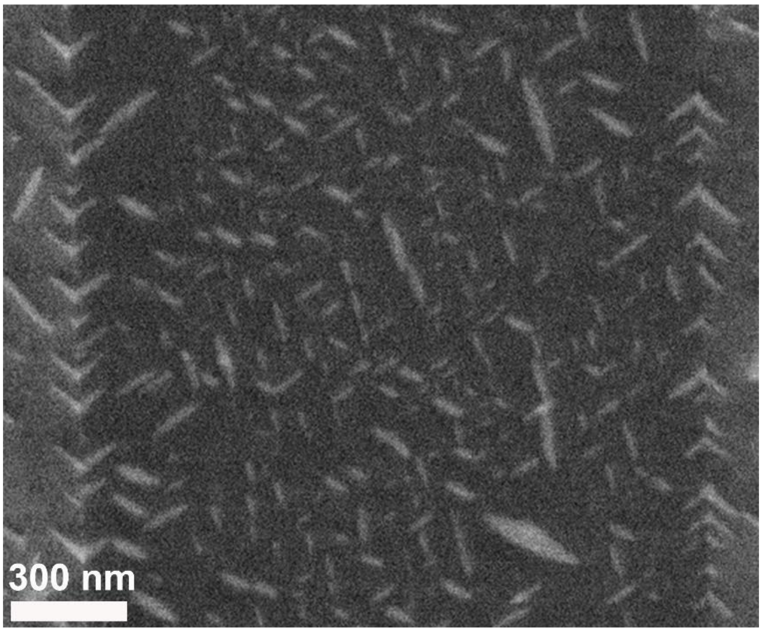


**Supplementary Figure 13.** BSE image of Inconel 718 after solution annealing at 1040 °C for 1 hour followed by cooling to room temperature with a cooling rate of 5 °C/min, and then aging at 802 °C for 8 hours.

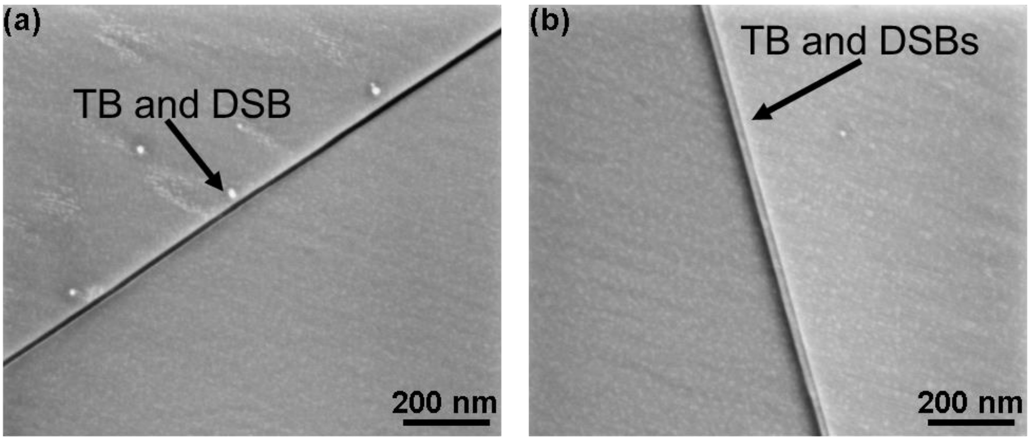


**Supplementary Figure 14.** BSE images showing γ″, TBs and DSBs in the sample after tensioning to a total strain of 1%. (a) a TB and a DSB very closely affiliated to the TB (hardly be separated) and (b) two parallel DSBs distributed very adjacent to a TB, in the under-aged 945X sample (annealed 1040 °C /1h, cooling in air; aged 706 °C/6 hrs + 607 °C/18 hrs)

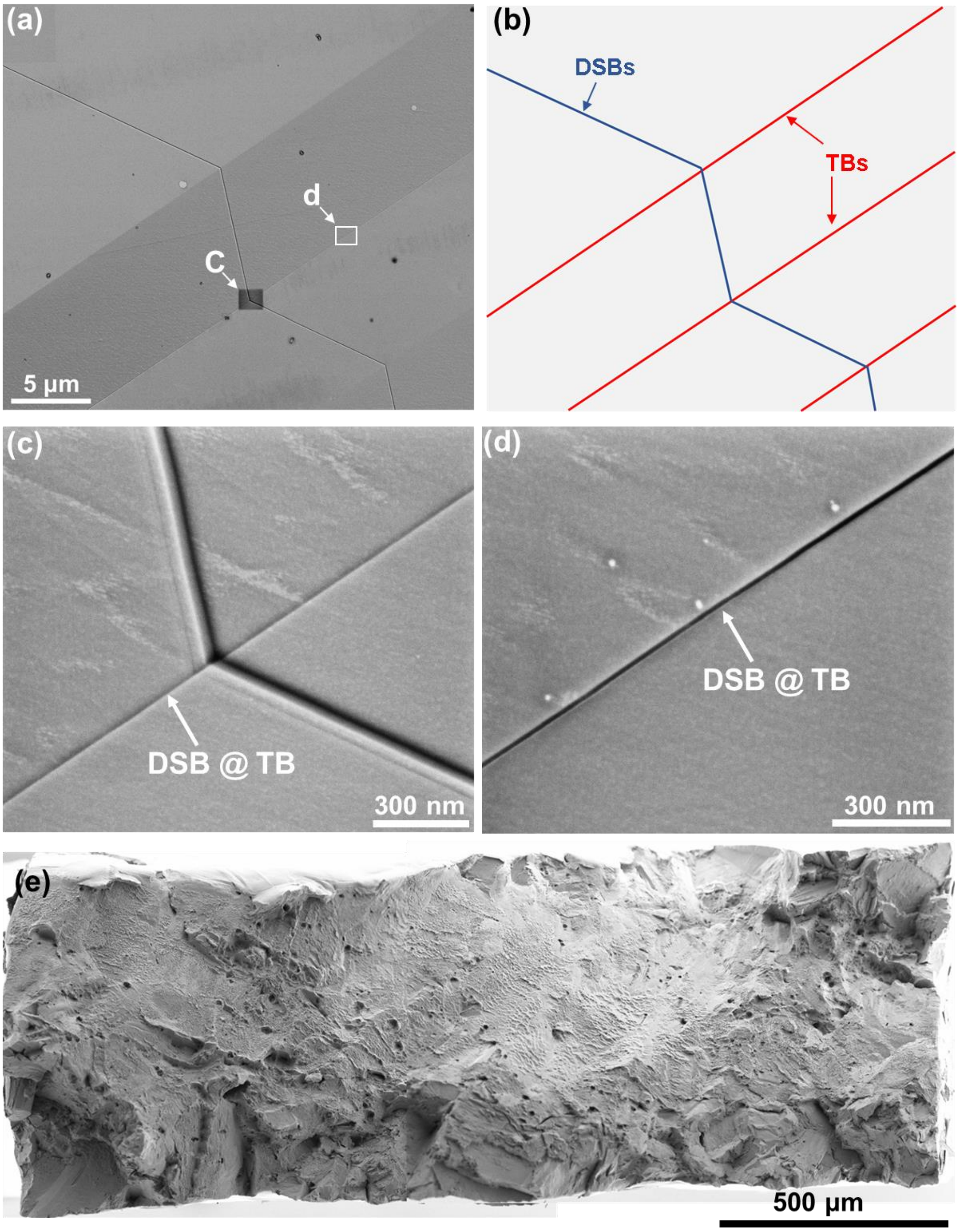


**Supplementary Figure 15.** Microstructural study on the dislocation slip bands (DSBs) and twin boundaries (TBs) in Alloy 945X-LB (low band treated)*: (a) BSE image shows DSBs across several TBs in the sample after tensioning to a strain of 1 %; (b) the schematic diagram of (a); (c) and (d) enlarged images from two regions marked in (a) showing DSB along a TB; (e) fracture surface of the H-charged sample after SSRT to failure. It can be seen from images (a)-(d) that the size difference in γ″ between that along TBs and in the grain interiors is not appreciable, which is very different to that observed in Fig.1 of 945X-HB (high band treated)*. Consequently, in contrast to 945X-HB, DSBs at TBs in 945X-LB are so close to TBs, so we cannot separate them even at very high magnification. It is also evident that DSBs are not only along TBs but also in grain interiors at this stage. Due to such significant difference in the character of TBs and dislocation plasticity between two samples, the fracture surface of H-charged

945X-LB is very different to that of 945X-HB. H-charged 945X-HB exhibits very flat facets on the fracture surface which results from cracking along TBs, whereas the fracture mode of 945X-LB is much more ductile and cracking along TBs is effectively suppressed.

* Alloy conditions:

| | |
|---|---|
| 945X-HB | 1040 °C /1h, 5°C /min cooling rate; 735 °C/6 hrs + 635 °C/18 hrs |
| 945X-LB | 1040 °C /1h, cooling in air; 706 °C/6 hrs + 607 °C/18 hrs |

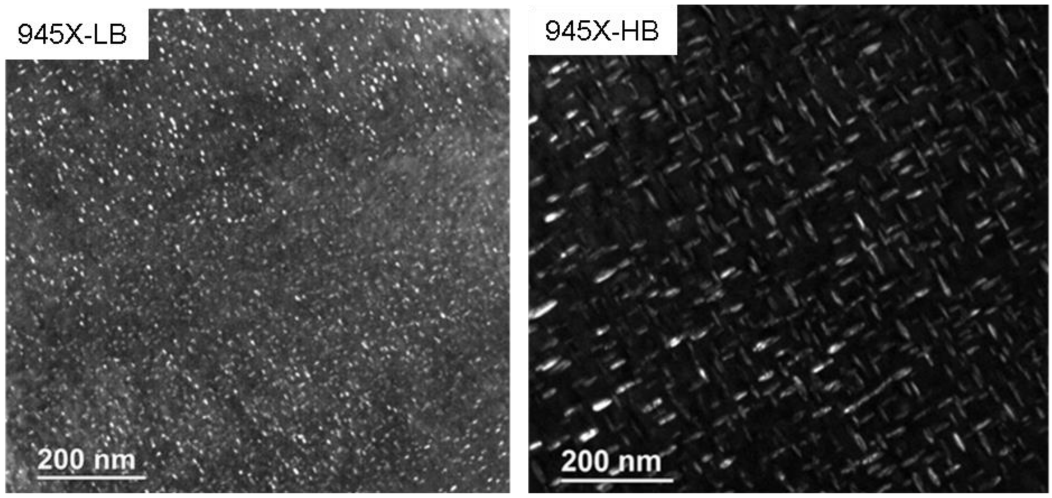


| | Non-Charged Elongation (%) | H-charged Elongation (%) | Deduction (%) | Yield strength (MPa) | $\sigma_{Twin}/\sigma_{Matrix}$ |
|---|---|---|---|---|---|
| 945X-LB | 39.3 | 18.2 | 53.6 | 922 | 0.90 |
| 945X-HB | 22.6 | 2.1 | 90.7 | 1150 | 0.68 |

**Supplementary Figure 16.** Microstructure and properties of 945X-LB and 945X-HB: (a) and (b) dark field TEM images along [100] zone axis showing the morphology of γ″ in the two samples; (c) properties of H-charged and non-charged alloys under the two conditions. It shows that 945X-LB has much smaller γ″ than 945X-HB, with average size of 16 nm and 39 nm respectively. As shown in the table, the H susceptibility of 945X-LB is much lower. In addition, using in-situ loading we obtained the stress level for DSBs initiation at TBs ($\sigma_{Twin}$) and grain matrix ($\sigma_{Matrix}$) in the non-charged sample. The ratio between them indicates how large of the γ″ effect on the dislocation activities at TBs. It can be seen that the $\sigma_{Twin}/\sigma_{Matrix}$ ratio is much larger in 945X-LB sample, which means the dislocation activities commence at similar stress level at TBs and grain interiors. In this manner, strain is more evenly distributed in the sample and the strain localisation at TBs is largely mitigated, which is believed to be the main reason for the better performance of 945X-LB with H in presence.

# Supplementary Note 1

***DFT calculations: lattice parameters of $\gamma''$ and $\delta$ $Ni_3Nb$ phases***

To get the equilibrium structure of the $\gamma''$ phase, the total energies E (*c*/*a*, *V*) as a function of the *c/a* and the volume (*V*) are calculated, then we obtain the optimized lattice parameters from the contour plot of E (*c*/*a*, *V*). For the $\delta$ phase, the volume is assumed the same as that of the $\gamma''$ phase [1], with this constrain, the optimized lattice parameters are obtained from E (*b/a*, *c/a*) contour. The lattice parameters of $\gamma''$ and $\delta$ Ni3Nb phases obtained by EMTO methods (Table S1) agree well with the previous theoretical results using the GGA and LDA functionals [2, 3] as well as the experimental results [14,15].

Supplementary Table 1 Calculated lattice parameters of $\gamma''$ and $\delta$ phases of $Ni_3Nb$ in comparison with the previous theoretical and experimental results. The lattice parameter of $\delta$ phase is obtained with the constrain that it has the same volume as the $\gamma''$ phase.

| $\gamma''$ phase | a(Å) | c(Å) | c/a | $\delta$ phase | a(Å) | b(Å) | c(Å) |
|---|---|---|---|---|---|---|---|
| EMTO | 3.67 | 7.49 | 2.04 | EMTO | 5.12 | 4.24 | 4.53 |
| LDA [2] | 3.58 | 7.33 | 2.05 | LDA [3] | 5.02 | 4.18 | 4.47 |
| GGA [2] | 3.66 | 7.48 | 2.04 | GGA [3] | 5.13 | 4.27 | 4.57 |
| Exp. [4] | 3.624 | 7.406 | 2.04 | Exp. [5] | 5.14 | 4.23 | 4.53 |

***DFT calculations: stacking fault and twin boundary energies***

The calculated SFEs are summarized in Table S2. As a benchmark, the $\gamma_{\rm isf}$ of pure Ni is calculated to be 151.1 mJ $m^{-2}$, which is in good agreement with the previous DFT (e.g., 155 mJ $m^{-2}$ ) [6] and experimental (125 mJ $m^{-2}$ ) results[7].

For the matrix with composition of $Ni_{56}Cr_{25}Fe_{19}$, $\gamma_{\rm isf}$ is calculated to be 21.1 mJ $m^{-2}$, which is consistent with the experimental results showing that both Cr and Fe additions in Ni drastically decrease the SFE [8]. The estimated experimental value [8, 9] for the present composition is about 35 mJ $m^{-2}$.

Supplementary Table 1 Calculated $\gamma_{isf}$ for fcc Ni and Ni-Cr25-Fe19 and for the D022 structure of $Ni_3Nb$ and $Ni_3(NbTi)$, in comparison with other theoretical results.

| Composition | Structure | $\gamma_{isf}$ (mJ m$^{-2}$) | $\gamma_{tw}$ (mJ m$^{-2}$) | Previous $\gamma_{isf}$ (mJ m$^{-2}$) |
|---|---|---|---|---|
| Ni | *fcc* | 151.1 | 75.6 | 155 [6] |
| $Ni_{56}Cr_{25}Fe_{19}$ | | 21.1 | 10.6 | 35 [8] |
| $Ni_3Nb$ | $D0_{22}$ | 6.9 | 3.5 | 2.3 [2] |
| $Ni_3(Nb,Ti)$ | | 1.2 | 0.6 | |

For $Ni_3Nb$, the calculated $\gamma_{isf}$ is 6.9 mJ m$^{-2}$, which agrees with the previous DFT result (2.3 mJ m$^{-2}$) [2] where the structural relaxation was considered. Alloying Ti is found to strongly decrease the $\gamma_{isf}$ to 1.2 mJ m$^{-2}$. Based on the universal scaling relationship, $\gamma_{tw} = \frac{1}{2}\gamma_{isf}$ [10], the twin boundary energies are estimated to be 3.5 mJ m$^{-2}$ for $Ni_3Nb$ and 0.6 mJ m$^{-2}$ for $Ni_3(Nb,Ti)$, respectively.

***DFT calculations: interfacial energy of γ/γ″***

The γ″-$Ni_3Nb$ phase has two types of {001} planes, one with 100%Ni and the other with 50%Ni +50%Nb. Thereby, there are two (001)γ//(001)γ″coherent interfaces and the averaged interfacial energy is calculated in the following. The interface supercell is composed of 8 layers of (001)γ and 8 layers of (001)γ″, which contains the above two interfaces due to the periodic boundary conditions. The underlying lattice parameters are taken as those of γ″, and the γ structure is relaxed accordingly. The interfacial distance is also relaxed. The total energy is denoted as $E_{16}^{\gamma/\gamma''}$. The reference bulk energies of γ and γ″ phases are calculated using the incremental method. The total energy of a supercell containing 8 layers of (001) γ″ and 6 layers (001) γ, $E_{14}^{\gamma/\gamma''}$, is used to calculate the reference bulk energy of γ phase (per atom) according to, $E_\gamma = (E_{16}^{\gamma/\gamma''} - E_{14}^{\gamma/\gamma''})/4$). The bulk energy (per atom) of γ″ phase is calculated by $E_{\gamma''} = (E_{16}^{\gamma/\gamma''} - E_{12}^{\gamma/\gamma''})/8$, where $E_{12}^{\gamma/\gamma''}$ is the total energy of a supercell containing 8 layers of (001)γ and 4 layers of (001)γ″. The averaged interface energy of two (001)γ//(001)γ″coherent interfaces is calculated by, $\sigma_{\gamma/\gamma''} = \frac{E_{16}^{\gamma/\gamma''} - 16E_\gamma - 16E_{\gamma''}}{2A}$, where $A$ is the interface area. The obtained interfacial energies for Ni/$Ni_3Nb$ at ferromagnetic and paramagnetic states are 145 and 112 mJ m$^{-2}$, respectively. For $Ni_{56}Cr_{25}Fe_{19}$/$Ni_3Nb$

at paramagnetic state, $\sigma_{\gamma/\gamma''}$ is 76 mJ m$^{-2}$. The above results are in nice agreement with previously reported value (95±17 [11], 90 [12], 52 [13] mJ m$^{-2}$).

## Supplementary References


1. Li, C.-X., *et al.* Lattice parameters and relative stability of α″ phase in binary titanium alloys from first-principles calculations. *Solid State Commun.* **159**, 70-75 (2013).
2. Lv, D. C., McAllister, D., Mills, M. J., Wang, Y. Deformation mechanisms of D022 ordered intermetallic phase in superalloys. *Acta Mater.* **118**, 350-361 (2016).
3. Dai, S., Liu, W. First-principles study on the structural, mechanical and electronic properties of δ and γ″ phases in Inconel 718. *Comp. Mater. Sci.* **49**, 414-418 (2010).
4. Cozar, R., Pineau, A. Morphology of y' and y" precipitates and thermal stability of inconel 718 type alloys. *Metall. Trans.* **4**, 47-59 (1973).
5. Sundararaman, M., Mukhopadhyay, P., Banerjee, S. Precipitation of the δ-Ni3Nb phase in two nickel base superalloys. *Metall. Trans. A* **19**, 453-465 (1988).
6. Nie, X., Renhui, W., Yiying, Y., Yumei, Z., Dingsheng, W. Calculations of stacking fault energy for fcc metals and their alloys based on an improved embedded-atom method. *Solid State Commun.* **96**, 729-734 (1995).
7. Anderson, P. M., Hirth, J. P., Lothe, J. *Theory of Dislocations*. Cambridge University Press (2017).
8. Unfried-Silgado, J., Wu, L., Furlan Ferreira, F., Mario Garzón, C., Ramírez, A. J. Stacking fault energy measurements in solid solution strengthened Ni–Cr–Fe alloys using synchrotron radiation. *Mater. Sci. Eng. A* **558**, 70-75 (2012).
9. Zhao, S., Stocks, G. M., Zhang, Y. Stacking fault energies of face-centered cubic concentrated solid solution alloys. *Acta Mater.* **134**, 334-345 (2017).
10. Jin, Z. H., Dunham, S. T., Gleiter, H., Hahn, H., Gumbsch, P. A universal scaling of planar fault energy barriers in face-centered cubic metals. *Scr. Mater.* **64**, 605-608 (2011).
11. Devaux, A., *et al.* Gamma double prime precipitation kinetic in Alloy 718. *Materials Science and Engineering: A* **486**, 117-122 (2008).
12. Schleifer, F., Holzinger, M., Lin, Y.-Y., Glatzel, U., Fleck, M. Phase-field modeling of γ/γ″ microstructure formation in Ni-based superalloys with high γ″ volume fraction. *Intermetallics* **120**, 106745 (2020).
13. Moore, I. J., Burke, M. G., Palmiere, E. J. Modelling the nucleation, growth and coarsening kinetics of γ″ (D022) precipitates in the Ni-base Alloy 625. *Acta Mater.* **119**, 157-166 (2016).
