# Supplementary material for: Strain localisation and failure at twin-boundary complexions in nickel-based superalloys
Source: Nat Commun. 2020 Sep 29;11:4890. doi: 10.1038/s41467-020-18641-z (PMC7524752; doi:10.1038/s41467-020-18641-z)
Supplement: Supplementary file 2 — Description of Additional Supplementary Files [file 41467_2020_18641_MOESM2_ESM.pdf]

Description of Additional Supplementary Files

File Name: Supplementary Movie 1

Description: Video recorded using a Keyence VHX-5000 optical microscope during in-situ tensile loading of a 945X alloy sample. The video was recorded with a frame size of 1600×1200 pixels at 15 frames per second during loading. The loading axis is horizontal. The initial strain rate is 1×10-5 s-1, and the video shown here is accelerated 240 times.
